# Selecting cases from nuclear families for case-control association analysis

**DOI:** 10.1186/1471-2156-6-S1-S105

**Published:** 2005-12-30

**Authors:** Rachael M Moore, Tracy Pinel, Jing Hua Zhao, Ruth March, Ansar Jawaid

**Affiliations:** 1 Research and Development Genetics, AstraZeneca, Alderley Park, Macclesfield, SK10 4TG, UK; 2 Department of Epidemiology and Public Health, University College London, Gower Street Campus, London WC1E 6BT, UK

## Abstract

We examine the efficiency of a number of schemes to select cases from nuclear families for case-control association analysis using the Genetic Analysis Workshop 14 simulated dataset. We show that with this simulated dataset comparing all affected siblings with unrelated controls is considerably more powerful than all of the other approaches considered. We find that the test statistic is increased by almost 3-fold compared to the next best sampling schemes of selecting all affected sibs only from families with affected parents (AF_aff_), one affected sib with most evidence of allele-sharing from each family (SF), and all affected sibs from families with evidence for linkage (AF_L_). We consider accounting for biological relatedness of samples in the association analysis to maintain the correct type I error. We also discuss the relative efficiencies of increasing the ratio of unrelated cases to controls, methods to confirm associations and issues to consider when applying our conclusions to other complex disease datasets.

## Background

Case-control association studies are regaining popularity in the challenge to identify markers conferring susceptibility to complex diseases. A sample of affected cases is compared to a sample of suitable controls to test for association between allelic variants and disease status. In the recent past, family-based association designs were advocated to protect against spurious associations arising from population substructure. However, such designs are 2- to 5-fold less efficient than using unrelated controls [1]. Furthermore, methods such as genomic control and structured association have since been developed to detect and account for population stratification. These methods rely on the premise that stratification would lead to differences in allele frequencies between two or more populations and that these differences could be detected by analyzing anonymous markers [2-4].

Further improvements in power can be obtained by including sibships with multiple affected sibs that are readily available from prior linkage studies [1]. Most of this gain is generally attributable to an increased allele frequency difference between related cases and unrelated controls. When the number of affected relatives increases, the expected allele frequency of the high-risk allele increases in the cases but remains the same in the unrelated controls. In contrast, the frequency of the high-risk allele also increases in the control group when related controls are used.

Where genotyping more than one sibling from a family is cost prohibitive, it may be useful to select the most informative sib for association analysis. A recent study used allele sharing to select the most informative sib from sibships of various sizes and found that choosing the sib showing the greatest allele sharing from each sibship increased the efficiency of case-control associations under a variety of genetic models [5].

When using related subjects in case-control studies the correlations among relatives must be accounted for in the statistical analysis to avoid an increase in type I error. A number of tests have been proposed that take account of the sampling of biologically related subjects in the variance of test statistic. Risch and Teng [1] propose a transmission disequilibrium test- (TDT) like statistic for sibling data; Slager and Schaid [6] advocate an adjusted trend test that allows cousin data to be used as well as sibling data; Bourgain et al. [7] suggest a quasi-likelihood trend test, particularly when cases are selected from complex inbred pedigrees.

Here we examine the efficiency of a number of strategies for selecting cases from nuclear families with multiple affected subjects and comparing with unrelated controls to identify a known Kofendrerd Personality Disorder (KPD) disease susceptibility marker on a region of chromosome 5. We examine the efficiencies of a number of case selection strategies including those proposed by Fingerlin et al. [5] and Risch and Teng [1]. The test statistic at the disease locus for each selection scheme is compared with the maximum test statistic we observed, and the number of other associated markers identified is also considered. We discuss the impact of over-sampling controls relative to cases and present approaches for confirming putative associations.

## Methods

### Data

The Genetic Analysis Workshop 14 (GAW14) simulated dataset was used for this analysis. A region of chromosome 5 was known to us to contain a susceptibility locus for KPD and was chosen for investigation. The actual disease locus was originally blinded from those doing the association analysis. However, it became clear from the analysis which marker was the true association and hence results are reported with reference to the known answer. Data packages 206–210 containing 100 markers were used. The Aipotu family dataset (001) with KPD affection status was initially used for the analysis. Unrelated control populations of various sizes (50, 100, 200, 400, and 1,000) were created by randomly combining control replication sets (replicates 001–020). The control data sets for each scheme contained approximately the same number of unrelated controls as affected cases, with different randomly selected controls used in each scheme.

### Case selection schemes

Seven case selection strategies were compared. We selected one affected sibling at random from each family (RF), all affected siblings from each family (AF), and all affected sibs from families with one or both parents affected (AF_aff_). We also selected sibs on the basis of linkage and allele sharing information. Families with evidence for linkage were defined as those with a multipoint linkage NPL score >0 in the chromosome 5 region (centered at D05S0172) (calculated using GENEHUNTER [8]). We employed schemes using one sibling selected at random from families with evidence for linkage (RF_L_) and all siblings from those families (AF_L_). The selection of siblings with the most evidence for allele-sharing was achieved by using the IBD probabilities for each family [5]. The schemes considered used one affected sib from each family with the most evidence for allele sharing (SF) and one affected sib with the most evidence for allele sharing in families with evidence for linkage (SF_L_). The schemes are summarized in Table 1.

### Comparison of selection strategies

Because some of the selection schemes include multiple siblings per family, the test statistic proposed by Risch and Teng [1] was used to test for single-nucleotide polymorphism (SNP) marker associations in the region, with the adaptation of using the average number of siblings per family. The test statistic is of the form , where  and  are the estimated allele frequencies of the SNP marker in the cases and controls, and  is the estimated standard deviation of the difference.

The test statistic is defined as



in which



The average number of affected sibs per family is denoted *r*, *n *is the number of families, and *u *represents the number of unrelated controls per family.  and  represent the number of marker alleles carried by case *i *and control *i*,, respectively. The test statistic is distributed as a chi-squared with 1 degree of freedom.

The test statistic was calculated for all of the markers in the region using SAS ^® ^(SAS version 8.2, SAS Institute Inc., Cary, NC, USA) and markers yielding *p *< 0.05 were considered further. To compare the efficiencies of the selection strategies the ratio of the test statistic at the disease locus for the given selection scheme to the maximum test statistic obtained (using all affected sibs and 5 times the number of controls, test statistic = 19.47) was used. The number of other markers in the region showing evidence of association (*p *< 0.05) to KPD but not identified as the causal variant was also noted. Linkage disequilibrium (LD) across the region was visualized using the program HELIXTREE [9]. The program TRANSMIT [10] was used to calculate the TDT test for the disease susceptibility marker.

## Results and discussion

### Identification of disease locus

The efficiencies of the seven selection schemes using equal ratios of cases to controls are shown in Table 1. The most efficient scheme was sampling all affected individuals (*n *= 233) (AF) and 5 times the number of controls, which gave a test statistic of 19.47. However, using an equal number of controls resulted in a mere 7% reduction in efficiency. The AF scheme was considerably more powerful than the any of the other selection strategies, with a greater than 2.5-fold increase in the test statistic than the next best approaches; AF_aff _(*n *= 118), SF (*n *= 100), and AF_L _(*n *= 110), with efficiencies of 33%, 29%, and 27%, respectively. The remaining approaches were particularly lacking in power with efficiencies in the range of 22% to 9%. The number of other positives (*p *< 0.05) identified was approximately the same across all of the selection schemes.

The higher efficiency of the AF scheme relative to the other schemes may be attributable to the larger sample size alone. However, others have shown that the gain in efficiency of such a design is due to an increase in the disease allele frequency in the case group rather than sample size [1]. In this study, the average number of affected siblings per family was 2.33 (range 2 to 7). It would be interesting to explore the impact of sampling a greater number of siblings per family but we were unable to do this due to the limitations of the simulated dataset. Table 1 shows the risk allele frequency for the case sampling schemes; the AF and AF_aff _schemes result in the greatest enrichment for the risk allele followed by AF_L_, SF, SF_L_, RF_L_, and RF. This ranking corresponds well with ranking on the difference in the risk allele between cases and controls (differences were observed in the risk allele frequency for each of the control groups as controls were selected randomly for comparing with cases from each of the selection schemes).

Although using all available cases from families may be preferable, from a genotyping perspective, this may not be feasible. When restricted to using only one affected sibling from each family we show that selecting the sib with the greatest allele sharing in a family results in a greater efficiency than randomly selecting an affected sib from each family, as observed by Fingerlin et al. [5].

Although these findings hold true for the specific genetic and phenotypic models used in the simulated data set, it is not clear how robust these findings are across a range of genetic models (e.g., allele frequency, dominance, epistasis, penetrance, LD between marker and causal variant(s)) and phenotypic traits (e.g., continuous and categorical). However, it is beyond the scope of this article to investigate the impact of these factors on the selection strategies considered here.

### Confirmation of the disease locus

The risk locus for KPD on chromosome 5, B05T4136, was identified in the Aipotu population. In addition to the disease locus a number of other markers were found to be statistically associated with KPD. We attempted to strengthen confidence in the association by using a number of methods described below.

**Figure 1:**
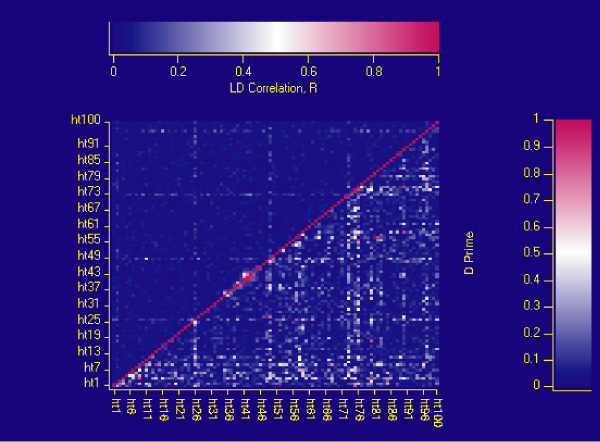
**Linkage disequilibrium plot for 95 SNPs across the KPD region**. The upper left triangle shows the pair-wise r values (labelled as LD correlation, R) and the lower right hand triangle shows pair-wise D' values (labelled d prime). Red indicates values of r or D' close to 1, blue represents values close to 0.

We examined the pair-wise marker LD across the region (Figure 1) using D' and *r *and found it to be low. This was not surprising given an average marker density of 1 per 0.3 cM. The known susceptibility marker was not found to be in strong LD with any of the markers genotyped in the region.

We next considered the potential existence of population stratification. We used a family-based TDT test of association to control for population stratification. The disease locus was found not to be associated, although a trend of increased transmissions of the risk allele was observed. Although the TDT is robust to population stratification, it is somewhat less powerful than case-control sampling [11]. The reduction in power is not only due to the smaller difference in allele frequency between cases and controls, as discussed above, but also because of the lower sample size. The reduction in sample size is attributable to only heterozygous parents being informative in the TDT, resulting in at least a third greater sampling and genotyping being needed compared to case-control sampling.

In addition to attempting to replicate the association in the same population, we also considered replicating in the available Karangar population. This marker was found not to be associated in the Karangar population. There are a number of reasons for not being able to replicate across populations. There could be important differences in allele frequency or LD structure across populations, resulting in the risk allele exhibiting a different pattern of association with marker alleles and haplotypes in the different populations [12]. Hidden population stratification can further complicate this situation by producing spurious association or changing the pattern of a true association [4,13-16]. Locus and allelic heterogeneity are also possible explanations.

## Conclusion

In the GAW14 simulated dataset we have shown that comparing all available cases from nuclear families with unrelated controls in an association study is considerably more powerful than any of the case selection schemes considered. However, strategies using all affected siblings with affected parents or cases with strong allele-sharing result in comparable enrichment of the risk allele but with fewer cases being selected. Although our results are similar to those published by other investigators, we suggest a degree of caution when generalizing all of these findings. Further investigation into the robustness of the results over a range of genetic and phenotypic models is required.

## Abbreviations

AF: All affected sibs selected from each family

AF_aff_ : All affected sibs selected from families with affected parents

AF_L_: All affected sibs from families with evidence for linkage

GAW14: Genetic Analysis Workshop 14

KPD: Kofendrerd Personality Disorder

LD: Linkage disequilibrium

RF: One affected sib selected randomly from each family

RF_L_: One affected sib selected randomly from families with evidence for linkage

SNP: Single-nucleotide polymorphism

TDT: Transmission disequilibrium test

SF: One affected sib with most evidence of allele-sharing from each family

SF_L_: One affected sib with most evidence of allele-sharing from families with evidence for linkage

## Figures and Tables

**Table 1:** Summary of each selection strategy

Scheme^a^	No. families	No. cases	No. controls	Allele freq cases	Allele freq controls	Difference allele freq	Test statistic	Relative efficiency (%)	No. other positive markers
RF	100	100	100	0.350	0.255	0.095	4.28	22	3
AF	100	233	200	0.384	0.232	0.152	18.20	93	7
AF_aff_	47	118	100	0.386	0.255	0.131	6.43	33	4
RF_L_	50	50	50	0.350	0.260	0.090	1.19	9	5
AF_L_	50	110	100	0.373	0.255	0.118	5.35	27	4
SF	100	100	100	0.365	0.255	0.111	5.66	29	4
SF_L_	50	50	50	0.360	0.260	0.100	2.33	11	4

^a^RF = One affected sib selected randomly from each family, AF = All affected sibs selected from each family, AF_aff _= All affected sibs selected from families with affected parents, RF_L _= One affected sib selected randomly from families with evidence for linkage, AF_L _= All affected sibs from families with evidence for linkage, SF = One affected sib with most evidence of allele-sharing from each family, SF_L _= One affected sib with most evidence of allele-sharing from families with evidence for linkage.

## References

[B1] Risch N, Teng J (1998). The relative power of family-based and case-control designs for linkage disequilibrium studies of complex human diseases. I. DNA pooling. Genome Res.

[B2] Devlin B, Roeder K (1999). Genomic control for association studies. Biometrics.

[B3] Satten GA, Flanders WD, Yang Q (2001). Accounting for unmeasured population substructure in case-control studies of genetic association using a novel latent-class model. Am J Hum Genet.

[B4] Pritchard JK, Stephens M, Rosenberg NA, Donnelly P (2000). Association mapping in structured populations. Am J Hum Genet.

[B5] Fingerlin TE, Boehnke M, Abecasis GR (2004). Increasing the power and efficiency of disease-marker case-control association studies through use of allele-sharing information. Am J Hum Genet.

[B6] Slager SL, Schaid DJ (2001). Evaluation of candidate genes in case-control studies: a statistical method to account for related subjects. Am J Hum Genet.

[B7] Bourgain C, Hoffjan S, Nicolae R, Newman D, Steiner L, Walker K, Reynolds R, Ober C, McPeek MS (2003). Novel case-control test in a founder population identifies P-selectin as an atopy-susceptibility locus. Am J Hum Genet.

[B8] Kruglyak L, Daly MJ, ReeveDaly MP, Lander ES (1996). Parametric and nonparametric linkage analysis: a unified multipoint approach. Am J Hum Genet.

[B9] HelixTree. http://www.goldenhelix.com/index.jsp.

[B10] TRANSMIT. http://www-gene.cimr.cam.ac.uk/clayton/software.

[B11] Cardon LR, Bell JI (2001). Association study designs for complex diseases. Nat Rev Genet.

[B12] Neale BM, Sham PC (2004). The future of association studies: gene-based analysis and replication. Am J Hum Genet.

[B13] Morton NE, Collins A (1998). Tests and estimates of allelic association in complex inheritance.. Proc Natl Acad Sci USA.

[B14] Thomas DC, Witte JS (2002). Population stratification: a problem for case-control studies of candidate-gene associations?. Cancer Epidemiol Biomarkers Prev.

[B15] Stumpf MP, Goldstein DB (2003). Demography, recombination hotspot intensity, and the block structure of linkage disequilibrium. Curr Biol.

[B16] Freedman ML, Reich D, Penney KL, McDonald GJ, Mignault AA, Patterson N, Gabriel SB, Topol EJ, Smoller JW, Pato CN, Pato MT, Petryshen TL, Kolonel LN, Lander ES, Sklar P, Henderson B, Hirschhorn JN, Altshuler D (2004). Assessing the impact of population stratification on genetic association studies. Nat Genet.

